# Qualitative evidence for the determinants of deaths of despair: a scoping review

**DOI:** 10.1186/s12889-026-27079-9

**Published:** 2026-03-21

**Authors:** Timothy Price, Amber Sacre

**Affiliations:** 1 Population Health Sciences Institute, Faculty of Medical Sciences, Newcastle University, Newcastle upon Tyne, NE1 4LP UK; 2 Newcastle Patient Safety Research Collaboration (PSRC), School of Pharmacy, Newcastle University, Newcastle upon Tyne, NE1 7RU UK

**Keywords:** Deaths of despair, Qualitative, Scoping review

## Abstract

**Background:**

Deaths from suicide, drug overdose, and alcohol-specific causes, collectively termed “deaths of despair” (DoD), represent a growing public health concern. Quantitative research has linked these deaths to adverse social and economic conditions but less is known about how their determinants are understood and articulated within qualitative research. This scoping review aimed to map the existing qualitative evidence on the perceived determinants of DoD.

**Methods:**

A scoping review was conducted in accordance with PRISMA-ScR guidelines. We searched CINAHL, Scopus, and Ovid Medline for qualitative studies published in English from 2015 onwards and conducted in OECD countries. Studies were included if they explored perceptions or beliefs related to the determinants of suicide, drug overdose, alcohol-specific mortality, or DoD collectively. Data were extracted and synthesised using a narrative thematic approach.

**Results:**

Of 3292 unique records identified, nine papers representing six distinct qualitative studies met the inclusion criteria. Studies were conducted in the United States, the United Kingdom, and Canada. Three overarching themes were identified: structural determinants (including economic insecurity, deindustrialisation, and limited access to services), social determinants (including weakened community cohesion, gender norms, stigma, and discrimination), and individual-level experiences (including psychological distress, hopelessness, and barriers to help-seeking).

**Conclusions:**

The qualitative evidence base on the determinants of deaths of despair is sparse and uneven. Existing studies situate DoD within broader structural and social contexts, highlighting the role of economic decline, service withdrawal, and social fragmentation in shaping pathways to distress, substance use, and self-harm. Further qualitative research across more diverse populations and settings is urgently needed to strengthen understanding of the mechanisms underpinning these deaths and to inform effective, upstream public health policy responses.

**Supplementary Information:**

The online version contains supplementary material available at 10.1186/s12889-026-27079-9.

## Introduction

Beginning in 2014-15, life expectancy in the United States (US), which had generally been increasing throughout the 20th and 21st centuries, began to stagnate and then decline [1]. Economists Anne Case and Angus Deaton attributed the decline in US life expectancy to increasing rates of death from drug, suicide, and alcohol-specific mortality among middle aged non-Hispanic Whites [2]. In their later work, Case and Deaton labelled these deaths as “deaths of despair” (DoD) [3, 4]. They proposed that shifting social norms (e.g. rising age at marriage, loss of communal identity), widespread poor health (e.g. chronic pain), and changes to the US labour market (e.g. low educational attainment, wage stagnation, and the cost of housing), had caused cumulative disadvantage from one birth cohort to the next and given rise to a sense of despair and ultimately increased the likelihood of DoD [3, 4]. Since Case and Deaton coined the term “deaths of despair” the phenomenon has gained greater recognition in both public and academic discourse [5–10].

While Case and Deaton’s [2, 3] work initially identified DoD as increasing specifically among middle aged non-Hispanic white Americans, more recent research has presented a more complex picture. In the United States (US), DoD are now more common among Black Americans than white Americans [11], with Native Americans and Alaskan Natives having the highest rates of DoD of any demographic group [12]. There is also growing recognition that DoD are not, as initially proposed, a uniquely American problem. Deaths from these causes have increased in recent years in the United Kingdom (UK) [13], and while rates have remained relatively stable elsewhere, DoD have been studied across a range of national contexts including Canada, Mexico, and former soviet countries in Eastern Europe [14–16]. Existing research on DoD remains geographically concentrated in high-income countries in the Global North, with only limited attention to low- and middle-income settings and particularly limited coverage of Africa, South Asia, and Latin America, highlighting an imbalance in the global application of the concept [17]. Deaths from these causes follow familiar patterns of health inequality. Across countries, DoD are concentrated in deprived, deindustrialised communities [6, 18, 19], occur in men at higher rates than in women [20, 21], and are more common among those with lower levels of educational attainment [3, 22].

While there is agreement that rates of DoD are influenced by broader social determinants of health, the existing literature pays limited attention to the root causes and social processes shaping DoD as well as the inequalities in DoD-related mortality across different social groups. As a result, scholars have yet to reach a consensus on whether DoD represents a distinct epidemiological pattern that warrants its own targeted research and intervention strategies [23, 24]. Beseran et al. (2022) conducted a scoping review of the quantitative, US based literature surrounding the determinants of DoD [25]. The review found that lower socioeconomic status, lower education levels, working in jobs with high insecurity, unemployment, and living in rural areas were the most relevant determinants of DoD, but that the literature surrounding the social determinants of DoD remains underdeveloped [25]. This review aims to expand on the 2022 scoping review by assessing what is known about the social determinants of DoD in the qualitative literature and in other geographic settings. In doing so, this review will further develop our understanding of the determinants of these deaths and identify areas for future qualitative investigation.

To our knowledge, no published work has reviewed qualitative studies exploring perceptions of the determinants of DoD, those due to drug, suicide, and alcohol-specific causes. This review aims to address this gap in the literature. In doing so, this review will map the available research on this topic, provide an overview of what is known thus far, identify whether the existing evidence is sufficient to support a systematic review and qualitative synthesis, and identify existing gaps in the literature.

## Methods

Our review protocol was registered with the Open Science Framework (Registration ID: FP2JU). Our review followed the Preferred Reporting Items for Systematic reviews and Meta-Analyses extension for Scoping Reviews (PRISMA-ScR) tool [26]. The PRISMA-ScR checklist for this review is included as supplemental material. The objective of this scoping review was to map the existing qualitative literature surrounding perceptions of the determinants of DoD and to identify gaps in the existing evidence base. Specifically, this review sought to ascertain:

1. How do members of the public and key stakeholders understand and explain the determinants of deaths of despair?
2. What cultural, structural, or community-level factors do qualitative studies identify as shaping deaths of despair?

### Search strategy and inclusion/exclusion criteria

We searched three online databases: CINAHL, Scopus, and Ovid Medline. Our search strategy was based on the approach of an existing scoping review of quantitative determinants of DoD [25], with changes made to capture qualitative, rather than quantitative literature, and studies that examined a single component of DoD (e.g. suicide) that did not use the term “deaths of despair”. The search strategy was designed for Medline and adapted to other database formats. Searches were piloted with two test papers to ensure the search strategy accurately returned relevant citations. Search strings were combined with date and country limits (Organisation for Economic Co-operation and Development (OECD) countries only, English-language publications from 2015 onwards) to ensure temporal relevance to current political and socioeconomic circumstances. As the term “deaths of despair*”* first entered common use in 2017 [3], this time frame also captures all literature produced after the concept gained recognition in academic and policy discourse. The review is limited to studies conducted in OECD countries to ensure comparability in socioeconomic contexts, health systems, and policy environments. Including non-OECD settings, where the social determinants of mortality differ markedly, would risk conflating distinct structural and cultural dynamics that shape health behaviours and outcomes. Studies were eligible for inclusion in this review if they met date and country restrictions, were published in English, used qualitative methods, and reported findings related to perceptions of the determinants of one or more causes of death in the DoD category (i.e. suicide, drug overdose, or alcohol-specific mortality). Searches were carried out in October 2025. The full search strategy and inclusion/exclusion criteria for this review are provided as supplemental material.

### Study selection, screening, and data extraction

**Fig. 1:**
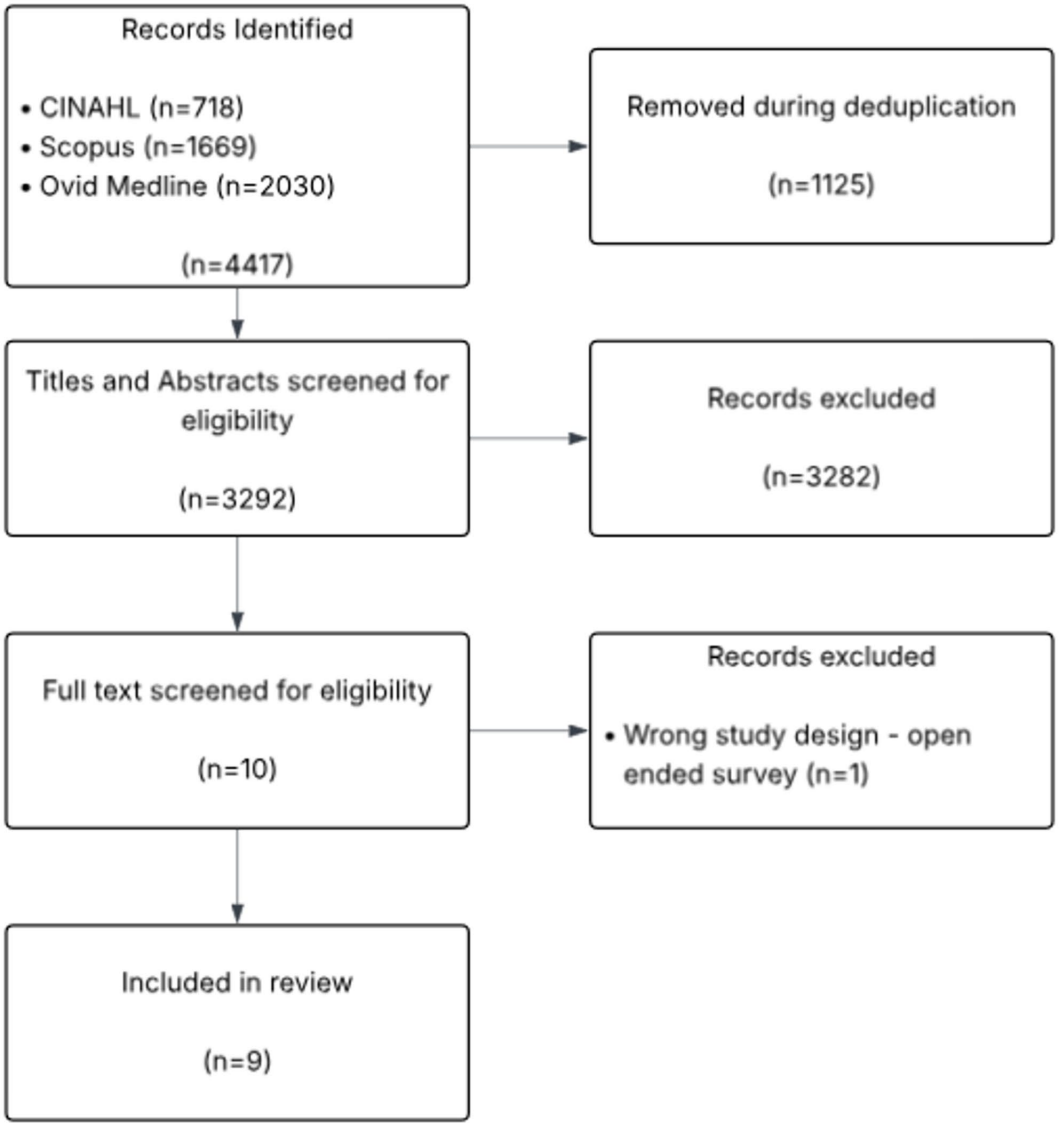
Inclusion process flow chart

One reviewer (TP) ran the initial searches to identify relevant articles. The title and abstract of all records were downloaded into Rayyan (https://rayyan.qcri.org/welcome) and deduplicated. Title and abstract screening, full-text screening, and data extraction were primarily conducted by a single reviewer (TP). To ensure consistency and reliability, a 10% sample of records at each stage underwent blinded double screening and data extraction by a second reviewer (AS). A high degree of agreement was reached between reviewers at each stage. Only one disagreement arose at the full-text screening stage regarding study inclusion; this was resolved through discussion, and the study was subsequently included. Reasons for exclusion were noted at the full-text stage. A bespoke data extraction form was used to extract relevant information from the selected studies. Information extracted included: Authors, year of publication, study setting, study design, participant details, method of analysis, and results. Figure 1 provides a flow chart of the inclusion process.

### Data synthesis

Extracted data were synthesised using a narrative, thematic approach. Following repeated reading of the extracted material, findings were compared across studies and three themes (structural, social, and individual determinants) were inductively generated. Particular attention was given to how participants in each study described the mechanisms linking broader social conditions to experiences of distress, substance use, and suicide. Themes were iteratively refined through constant comparison across studies, with divergences noted where findings differed by national context, population group, or cause of death.

## Results

### Characteristics of included studies

Nine papers were included in this review. Five papers reported findings related to the determinants of “deaths of despair” [32–35] or “diseases of despair” [28], two reported determinants of suicide [27, 31] and two reported determinants of drug overdose [29, 30]. None of the included papers reported findings only related to the determinants of alcohol-specific mortality. The included papers represent studies conducted in the UK [31–35], Canda [27], and the US [28–30]. Four papers report different findings derived from the same dataset or subsample of the same dataset [32–35]; thus, the included papers represent six distinct qualitative studies. A summary of each included paper is provided in Table 1.

**Table 1:** Characteristics of included studies

Study	Location	Cause of death studied	Sample size	Sample demographic details	Data collection and analytic method	Principal Findings
(Affleck et al., 2022) [27]	Nunavik, Canada	Suicide	18	Race: 18 Inuit. Age: 22–63 years old. Gender: 17 women, 1 man.	Interviews. Thematic analysis.	Intergenerational trauma from the Dog Slaughter, rapid socioeconomic change, and structural disadvantages have collectively undermined Inuit masculine roles, increasing risk of suicide and contributing to cycles of violence, marginalization, and poor mental health among boys and men.
(George et al., 2021) [28]	Pennsylvania, USA	Drug, suicide, alcohol-specific mortality	60	Race: 40 white, 17 Black, 3 Hispanic/Latino. Age: Not Provided. Gender: 44 women, 16 men.	Focus groups. Thematic analysis using a descriptive phenomenological approach.	Community members linked despair-related illness to long-term social and economic decline exacerbated by financial strain, weak infrastructure, and fragmented communities.
(Mclean, 2016) [29]	Pennsylvania, USA	Drug Related Mortality	18	Race: 13 white, 5 Black. Age: Majority under 40. Gender: 6 women, 12 men.	Interviews. Thematic analysis.	Overdose risk was shaped by unsafe private injection environments, minimal harm-reduction knowledge or resources, and a broader community context of economic decline, pervasive poverty, drug saturation, and social disintegration that left residents hopeless and vulnerable to fatal drug use.
(Thompson et al., 2020) [30]	Pennsylvania, USA	Drug Related Mortality	20	No demographic details provided.	Interviews. Thematic analysis.	Opioid use was reinforced by family and peer norms, community-wide visibility and media dynamics, scarce youth and neighbourhood resources, economic decline, weak community cohesion, and pervasive hopelessness, all of which foster boredom, disengagement, and reliance on drugs as coping and social mechanisms.
(Marzetti et al., 2022) [31]	Scotland, UK	Suicide	24	Race: Not Provided Age: 16–24 years old. Gender: 11 women, 7 men, 6 outside of gender binary.	Interviews. Reflexive thematic analysis.	Participants described how pervasive cis-heteronormativity, queerphobic bullying, and family or community rejection created a sense of “queer entrapment,” in which exploring LGBT+ identity felt emotionally unsafe, leading some to view suicide as the only escape.
(Price, 2024) [32]*	North East England, UK	Drug, Suicide, alcohol-specific mortality	54	Race: Not Provided. Age: 18–65+ Gender: 23 women, 31 men.	Interviews. Thematic analysis.	Participants linked austerity-driven cuts to mental-health services, youth provision, and community infrastructure with rising isolation, substance use, and hopelessness, which reinforced worsened well-being and increased risk of DoD.
(Price et al., 2024) [33]*	North East England, UK	Drug, Suicide, alcohol-specific mortality	13	Race: Not Provided Age: 25–64 Gender: 7 women, 6 men.	Interviews. Thematic analysis.	Participants attributed DoD to deep-rooted structural deprivation, driven by deindustrialisation, austerity, and poor housing, combined with normalized drug use and a fractured local identity.
(Price, 2025a) [34]*	North Eat England, UK	Drug, Suicide, alcohol-specific mortality	54	Race: Not Provided. Age: 18–65+ Gender: 23 women, 31 men.	Interviews. Thematic analysis	Moralisation, territorial stigma, and reliance on downstream interventions obscures the structural determinants of DoD and thereby sustains entrenched geographic inequalities in post-industrial England.
(Price, 2025b) [35]*	North East England, UK	Drug, Suicide, alcohol-specific mortality	54	Race: Not Provided. Age: 18–65+ Gender: 23 women, 31 men.	Interviews. Thematic analysis.	Participants linked high male DoD rates to traditional working-class masculine norms that persist despite deindustrialisation, leaving men unable to meet breadwinner expectations, unwilling to seek help due to masculine stoicism, and more likely to cope through substance use, violence, and self-harm.

*These papers report different findings derived from the same dataset (or subsample of the same dataset)

### Structural determinants

In five of the included studies, participants linked DoD to structural disadvantage, namely poverty, lack of economic opportunity, and a retrenchment of public services [27–31]. A central component of the structural disadvantage described by participants in these studies was the long-term impacts of deindustrialisation and accompanying economic decline. Participants in these studies described how regions (Appalachia in the US and North East England in the UK) once characterised by stable employment in manufacturing, resource extraction, and other traditionally male-dominated sectors, were now marked by poverty, insecure and low-paid work, and diminished future prospects [27–31]. Deindustrialisation in these regions was seen to have eroded not only material security but also the social status and sense of purpose previously attached to local forms of labour. Participants in one study described men feeling “left behind” as economic restructuring privileged different skills and identities, contributing to widespread demoralisation and intensifying vulnerability to substance use and self-harm [31]. In another study, participants described how the level of poverty in Appalachian Pennsylvania was so severe that people had no reason not to engage in drug use [28].

“Well, I think a lot of it is the, the income rate of people around here. It’s so low income. The people don’t have, like, stuff, so they turn to drugs easier when you’re poor. At least for me it was easier for me to turn to drugs, because I didn’t really have anything in my life to lose…” (McLean, 2016, pg. 24) [28].

Compounding this economic marginalisation was a lack of available essential services. Participants in five studies described shrinking mental and physical healthcare infrastructure, long wait times, and limited access to appropriate or culturally relevant support, particularly for men. Participants across geographic settings felt that the lack of available services left people without support when struggling with their mental health, increasing the risk of suicide [29–32, 33].

“Some people do need access to mental health care because I know, people among work and my own family, … if they want to schedule appointments with mental health providers, it’s like, well, this is 2 months in advance. So, really disheartening to wanna reach out and you’re willing to get help but just can’t get it.” (George et al., 2021, Pg. 6) [27].

The lack of services extended beyond healthcare into youth services, community centres, and social programmes, with some participants reporting the absence of virtually any preventive or recreational resources in their communities. The reasons for the inaccessibility/absence of services varied between settings; in the US, the market-based healthcare system and rural nature of Appalachian communities was to blame [27–29], in Canada it was the rurality of Inuit communities and the school-based nature of existing services that reduced access [32], and in the UK services had been cut due to austerity measures [30, 33].

In the context of Inuit communities in Canada, the structural determinants identified in one study included the legacy of colonisation and resulting intergenerational trauma. Participants described the lasting social and psychological impacts of events such as the Dog Slaughter and other forms of state violence, which disrupted cultural continuity, diminished traditional roles, and weakened mechanisms of community governance and intergenerational care [32]. These historic injustices were seen to shape contemporary patterns of harm, contributing to cycles of substance use, violence, and suicide as younger generations navigated identities destabilised by both cultural loss and ongoing socioeconomic marginalisation [32].

### Social determinants

The included studies also highlighted a set of cultural and community-level forces that shaped pathways into self-harm and substance use. Central among these was the role of gender norms and shifting expectations around masculinity. In four of the included papers, men described a profound sense of identity loss as structural changes (such as deindustrialisation and the rise of unstable work) undermined their ability to fulfil culturally valued roles as providers, protectors, or workers [28–32, 31]. This erosion of masculine identity left many men in deindustrialised communities in the US and UK feeling ashamed, useless, or emasculated, contributing to emotional distress and reluctance to seek help.

“Secondly, we’ve still got that old expectation that men should provide. You were brought up with the “mens should be the provider” type of thing and then if you’re not able to be the provider you lose your self-worth.” (Price, 2025, pg. 6) [31].

A second major social determinant was the fragmentation of community cohesion. Participants in four of the included papers described a decline in neighbourliness, trust, and/or informal mutual assistance, as communities weakened under the pressures of economic precarity, population turnover, and social disinvestment [27–30]. In two studies [27, 29], older residents of Appalachia contrasted contemporary isolation with earlier periods when families and neighbours actively supported one another, while younger people reported limited opportunities for connection and few trusted adults in their lives.

“If you’d ask my next-door neighbor my son’s name, what he wanted to do, where he wanted to go, they’d have no idea. Because we don’t talk to each other or help each other like we used to. And if we think decades back, we hung out with neighbors every night…. It [isolation] impedes people asking for help.” (George et al., 2021, pg. 7) [27].

These dynamics were further shaped by intergenerational cycles of trauma and disadvantage. In two studies that represented both Indigenous and non-Indigenous contexts, participants described growing up in families marked by violence, instability, untreated mental-health problems, or entrenched substance use, with few positive role models or stable sources of care [29, 32].

“Men here are all fucked up. It’s not just the suicides, it’s the crazy drinking, the violence against women, their morals are fucked because they had nobody to guide them … It’s a learned thing. They were raised by broken men who were also raised by broken men.” (Affleck et al., 2022, pg. 3) [32].

Social norms surrounding substance use were identified as determinants of substance use in four studies. Drug use, particularly opioids and marijuana, was described as pervasive, socially accepted, and often seen as “normal” within peer and family networks [28–31]. Such environments were seen to create strong pressures toward initiation and made abstaining or recovering extremely difficult. Participants in these studies emphasised that when “everyone uses,” substance use becomes both a social activity and a shared coping mechanism, with limited alternative ways to belong or manage distress. Boredom and lack of meaningful activities were repeatedly cited as drivers of substance use by participants in the US and UK.

“There is no sense of community here. Not one, not one iota of community here. Not one. So, left to your own devices, somebody that’s drinking and drugging is gonna continue drinking and drugging. Nothing else, cause there ain’t shit else to do.” (McLean, 2016, pg. 25) [28].

In two studies, participants noted that the harms associated with substance use only became visible or politically salient when they affected more privileged groups in other places, reinforcing marginalised communities’ sense of neglect and eroding trust in public institutions [28, 29]. Relatedly, Price (2025a) identified that moralising narratives in media, territorial stigma, and reliance on narrow, downstream interventions similarly shaped public perceptions of DoD, eroded local trust in institutions, and led to feelings of alienation and betrayal in affected communities. Participants in that study felt these factors obscured the structural drivers of distress, framed substance use as a personal or moral failing, and reinforced the social devaluation of post-industrial communities in North East England [34].

“When you mention you’re from Middlesbrough you get “oh smoggies.” That’s a nickname for people from Middlesbrough back when it was very industrial with all the smoke. It’s seen as a really deprived place. It’s been on some quite nasty TV shows where people were portrayed really negatively. It’s called Benefits Street, and it was in Middlesbrough. Stockton as well. So, we get a lot of negative portrayal. A few years ago one of the newspapers had Middlesbrough as the worst place in the UK to live.” (Price, 2025a, pg. 3) [34].

In the one study reporting data on LGBTQ+ communities, participants felt that queerphobia significantly contributed to the risk of self-harm and suicide. Participants in this study experienced queerphobia from family members, healthcare providers, and from society at large. These participants reported that repeated experiences with queerphobia lead to feelings of dehumanisation and isolation [35].

### Individual determinants

Six of the included studies also provided evidence surrounding individual-level experiences and behaviours that translated into personal risk for self-harm and substance use. Participants in these studies described how economic precarity resulted in severe distress which manifested as feelings of hopelessness, purposelessness, and emotional exhaustion [29–32]. In three of these studies, participants felt that substance use and self-harm emerged as a coping mechanism that managed psychological pain, served as an escape from traumatic memories, or provided temporary relief from overwhelming stress [28–30].

“It seems like with the economic environment, family situations, violence, gang violence, there is a lot of psychological impacts that it’s having on families. And my assessment would be that how people cope with them is in different ways. And for some people, it’s using substances.” (Thompson et al., 2020, pg. 5) [29].

Difficulty engaging with support systems was also identified as an individual determinant in four of the included studies [28–32, 35]. Participants in these studies described significant barriers to help-seeking, including long wait times, distrust of services, previous negative experiences, and perceptions that available programs were not designed for people like them, particularly for men, Indigenous people, and members of the LGBTQ+ community [28–32, 35].

Finally, two studies identified one’s immediate lived environment, particularly their housing, as a determinant of substance use. McClean [29] found that individuals living in precarious or unsafe accommodation in Appalachia, including hostels, temporary lodgings, or informal arrangements, were routinely exposed to environments where drug use was normalised, often unavoidable, and particularly dangerous. Participants described housing settings saturated with substance use, where the presence of drugs, dealers, or peers who were actively using made abstinence extremely difficult and created constant pressure to participate. Housing precarity also forced many to use drugs in unsafe, hidden spaces to avoid eviction or police attention, increasing overdose risk and reducing the likelihood of timely intervention. Price et al. similarly found that in North East England, unstable or low-quality housing diminished individuals’ capacity to stabilise their lives, maintain routines, or distance themselves from harmful networks while in recovery [30].

“The private rented sector especially but even some of the hostels and registered providers, some of the social landlords, I sort of, people who are placed in those accommodation options are surrounded by active drug use, drug dealing, bothering them, tempting them, manipulating them, crime, antisocial behaviour, all of these things … they’re dangerous places. Not the sort of place you’d want to live.” (Price et al., 2024, pg. 5) [30].

## Discussion

This scoping review has synthesised qualitative literature on the determinants of DoD across three cultural and geographic contexts, the US, UK, and Canada. Three interconnected categories of determinants, structural, social, and individual, were present in the included literature, with structural forces appearing most prominently and consistently across the studies. Although we have presented structural, social, and individual determinants as distinct themes in our analysis, these are heuristic categories rather than mutually exclusive classifications, and there is a degree of overlap between them. Participants described how long-term economic decline, particularly in deindustrialised communities, and the erosion of stable labour markets had reshaped the social fabric of their respective areas. Loss of industry and secure employment was reported to have led not only to material hardship and reduced future prospects but also to diminished social status, purpose, and identity. These forms of structural disadvantage intersected with shrinking or inaccessible public services, especially mental health care, substance use treatment, and community supports, intensifying distress and limiting available pathways for support. Social determinants, including weakened community cohesion, shifting or unattainable expectations surrounding masculinity, and intergenerational trauma, further amplified individual vulnerability. Finally, the reviewed studies identified individual-level experiences such as psychological distress, hopelessness, trauma histories, and difficulty engaging with services as proximal contributors to substance use and self-harm.

These finding are supported by the existing public health and health inequalities literature. Deindustrialisation and the resulting economic distress have been identified as significant determinants of health and driving forces underlying regional health inequalities [19, 36–38]. That the retrenchment of public supports and lack of access to healthcare and harm reduction services was identified as determinants of DoD is similarly inline longstanding evidence on the centrality of welfare infrastructures, service accessibility, and social protection for population health [39–41]. In this sense, the qualitative DoD literature largely aligns with broader public health and health inequalities scholarship: the same structural conditions that are known to erode health (e.g. economic insecurity, labour market precarity, service withdrawal, and widening social inequalities) also appear to shape the pathways through which substance use, self-harm, and ultimately DoD occur. In light of this literature, the findings of this review suggest that rather than representing a wholly unique phenomenon, DoD can therefore be understood as an acute manifestation of well-established social determinants of health operating in particularly concentrated and harmful ways.

The findings of this review largely echo those of an existing scoping review of quantitative literature examining the determinants of DoD, which identified low socioeconomic position, working in jobs with high insecurity, unemployment, and living in rural areas as the most relevant determinants of DoD [25]. Our review suggests that qualitative research can complement this body of work by exploring how such structural forces are experienced in everyday life and how they are understood to translate into substance use and suicide across a range of communities. However, only nine qualitative studies met our inclusion criteria, four of which [28–35] examined just one component of DoD and four of which [30, 31, 33, 34] were derived from the same underlying dataset. The qualitative evidence base therefore remains small and uneven. Further qualitative research across a wider range of populations and settings would expand the DoD literature by broadening our understanding of the lived experiences and social processes that contribute to DoD.

Despite its basis in a small body of literature, this review has identified intersectional dimensions of DoD determinants. Intersectionality is a theoretical framework that examines how multiple social categories, such as race, gender, class, and sexuality, interact to produce distinct and mutually reinforcing forms of privilege and oppression [42]. Intersectionality has been applied widely in public health research to identify how structural inequities shape health risks and outcomes across different groups [43–45]. Although only two studies explicitly examined DoD determinants within specific minoritised groups [32, 35], their findings demonstrate that the individuals with minoritised identities encounter additional and qualitatively different determinants, such as queerphobia [35] and legacies of colonialism [32], compared with non-minoritised groups, that may exacerbate their risk of DoD. This finding is of particular importance, given the disproportionately high rates of DoD experienced by some minoritised groups, such as members of the LGBTQ+ community [46, 47], Indigenous people [48, 49], and, in recent years, Black Americans [11, 50]. Future qualitative research should explore DoD within a broader range of social groups, identities, and geographic contexts with a specific emphasis on reaching marginalised groups. Given the disproportionately high burden of DoD experienced by these groups, qualitative work that captures their specific social, historical, and cultural contexts is critical. Intersectional, community-led, and participatory approaches would be particularly valuable in exploring how multiple forms of disadvantage interact to shape vulnerability to DoD [51].

This review also speaks to an ongoing debate in the literature concerning the conceptual value of “deaths of despair” as a unified category. Some scholars contend that grouping suicide, overdose, and alcohol-related mortality risks oversimplifying distinct phenomena with unique structural, cultural, and biomedical pathways [23, 52–54]. Others argue that these causes of death share common structural drivers and therefore benefit from being examined together [13, 24, 55]. The qualitative evidence identified here provides partial support for the latter perspective: despite differences across national contexts and regardless of whether a given study investigated one cause of death or DoD more broadly, participants consistently described similar mechanisms linking structural disadvantage and social marginalisation to suicide and deaths related to substance use. However, the findings also indicate there may be heterogeneity introduced by identity, historical context, and cultural experience, particularly for Indigenous and LGBTQ+ populations. It is also important to note that the qualitative literature captured in this review did not include any studies conducted in low- and middle-income countries, and such settings remain significantly underrepresented in the DoD literature [17]. This limits our ability to assess the conceptual utility of the DoD label beyond high-income contexts in which it was originally developed and has thus far been applied. Thus, while the DoD framework may be a useful heuristic for drawing attention to structural harms, it should be applied with caution, ensuring that it does not obscure diverse lived experiences or neglect important distinctions between different forms of mortality. Future qualitative research could provide further insight into the utility of grouping drug, suicide, and alcohol-specific mortality together by examining whether and how individuals and communities understand DoD as a distinct phenomenon. Such research would provide greater insight into how these deaths are understood by members of the public and would help to clarify whether the DoD framework is analytically and empirically appropriate.

### Strengths and limitations

This review has several strengths. It is, to our knowledge, the first study to systematically gather and synthesise qualitative research on the determinants of DoD, providing interpretive depth to an area of growing empirical and policy interest. It complements an existing quantitative review and in gathering qualitative evidence has provided insight into the lived, relational, and contextual dimensions of DoD that are not easily captured in studies using quantitative data [25]. Methodologically, the review was conducted in line PRISMA-ScR, which has ensured that the review was conducted to a rigorous standard.

However, the review also has important limitations. This review did not include a formal assessment of study quality; as a result, the strength or robustness of the included evidence cannot be evaluated. Despite using broad search parameters, the evidence base we found is extremely limited; only nine studies met the inclusion criteria, and four [28–35] examined only one component of DoD, of which none examined determinants of alcohol-specific mortality. Furthermore, four of the included papers [30, 31, 33, 34], though reporting distinct analyses, derived from a single dataset, meaning the review ultimately reflects findings from only six unique qualitative studies. The majority of the included studies did not provide detailed demographic details of their participants, making it difficult to ascertain whether the existing evidence base adequately reflects the diversity of populations affected by DoD. The majority of the included evidence was collected in two geographic settings, North East England and Pennsylvania, both of which have similar experienced significant deindustrialisation and resulting deprivation [56–59]; it is possible that people in different settings view the determinants of DoD differently. Given these limitations, the primary conclusion supported by this review is therefore not a definitive statement about the determinants of DoD but rather the observation that qualitative research in this domain is sparse, uneven, and urgently needed. Future qualitative studies across a wider range of settings, populations, and local contexts are necessary to build a more complete and nuanced understanding of DoD and to support the development of responsive, evidence-informed interventions.

## Conclusion

This scoping review set out to map the qualitative evidence on how members of the public and key stakeholders understand and explain the determinants of DoD. Across the nine included studies, participants linked suicide-, drug-, and alcohol-specific mortality to the convergence of structural disadvantage, social fragmentation, and individual distress. Structural determinants, including deindustrialisation, economic insecurity, and inaccessible services were the most prominent themes shaping pathways into despair. Social determinants, such as weakened community cohesion and pervasive substance-use cultures further compounded this disadvantage. At the individual level, participants highlighted hopelessness, psychological pain, and difficulties accessing or trusting support systems as immediate drivers of self-harm and substance use. These findings suggest that DoD are understood not as isolated personal crises but as outcomes of intersecting structural and social conditions that erode wellbeing and limit avenues for coping.

Our review also found substantial gaps in the qualitative evidence base. Despite growing research and policy interest in DoD, very few qualitative studies have explored how people themselves make sense of these deaths or the broader contexts in which they occur. This scarcity of qualitative literature presents important opportunities for future research. Qualitative research is well positioned to explore how structural determinants are experienced in everyday life, how communities understand and respond to distress, and how identities, histories, and local contexts shape vulnerability and resilience to these deaths. Richer qualitative inquiry could deepen our theoretical understandings of DoD and support the development of more responsive, contextually grounded interventions. Future research should engage diverse populations, employ intersectional and community-led approaches, and aim to capture the complex social processes through which despair is produced and navigated. Such qualitative investigation will allow us to develop a fuller, more nuanced understanding of DoD and inform policies capable of addressing their structural and social roots.

## Supplementary Information

Supplementary Material 1.

Supplementary Material 2.

## Data Availability

The datasets used and/or analysed during the current study are available from the corresponding author on reasonable request.
