# Supplementary material for: Qualitative evidence for the determinants of deaths of despair: a scoping review
Source: BMC Public Health. 2026 Mar 21;26:1400. doi: 10.1186/s12889-026-27079-9 (PMC13127069; doi:10.1186/s12889-026-27079-9)
Supplement: Supplementary file 2 — Supplementary Material 2. [file 12889_2026_27079_MOESM2_ESM.docx]

| **Concept** | **Search Terms** |
| --- | --- |
| Deaths of Despair | Death* of despair OR disease* of despair OR suicid* OR self-harm* OR self-injur* OR suicid* attempt OR drug addict* OR overdose* OR drug poisoning* OR drug user* OR SUD OR AUD OR alcoholi* OR alcohol related death OR alcohol specific death |
| **AND** | |
| Qualitative Research | Qualitative OR qualitative analysis OR qualitative research OR participatory OR ethnograph* OR phenomenolog* OR feminis* OR narrative* OR Interview* OR focus group* OR case stud* OR anthrop* OR thematic OR constant comparative OR observ* OR field notes |
| **AND** | |
| Determinants | Determinant* OR social determinants of health OR driv* OR health inequality* OR health disparit* OR health inequit* |

*Supplementary Table 1: Search strategy used in this review. This search strategy is based upon the search strategy developed by Beseran et al. (2022).*

| **Inclusion:** | **Exclusion:** |
| --- | --- |
| Studies reporting primary data of any qualitative design, for example: ethnographic studies, studies that used a phenomenological or grounded theory approach, or participatory action research. | Unpublished data, abstracts, conference proceedings, grey literature, and studies including no primary data. |
| Studies published in English. | All studies using quantitative methods, as well as studies that use self-report or researcher-administered surveys, including those which attempt to analyse data from open-ended questions, as the sole method of data collection,  Qualitative research that has not ascertained lay perspectives but has analysed texts e.g. discourse analysis. |
| Studies that explore perceptions of the determinants of deaths of despair, (or deaths resulting from one or more of the following: suicide, alcohol abuse, drug overdose), by members of the public or stakeholders. | Studies not published in English. |

*Supplementary Table 2: Inclusion and exclusion criteria.*
